# Ultra-Mild Fabrication of Highly Concentrated SWCNT Dispersion Using Spontaneous Charging in Solvated Electron System

**DOI:** 10.3390/nano14131094

**Published:** 2024-06-26

**Authors:** Junho Shin, Jung Hoon Kim, Jungeun Lee, Sangyong Lee, Jong Hwan Park, Seung Yol Jeong, Hee Jin Jeong, Joong Tark Han, Seon Hee Seo, Seoung-Ki Lee, Jungmo Kim

**Affiliations:** 1 Nano Hybrid Technology Research Center, Korea Electrotechnology Research Institute (KERI), Changwon 51543, Republic of Korea; sinjh@keri.re.kr (J.S.); kjh40210@keri.re.kr (J.H.K.); eeueu@keri.re.kr (J.L.); tkddyd@keri.re.kr (S.L.); jhpark79@keri.re.kr (J.H.P.); syjeong@keri.re.kr (S.Y.J.); wavicle11@keri.re.kr (H.J.J.); jthan@keri.re.kr (J.T.H.); seosh@keri.re.kr (S.H.S.); 2 School of Material Science and Engineering, Pusan National University, Busan 46241, Republic of Korea

**Keywords:** carbon nanotubes, dispersion, solvated electron, charging, high concentration, defect free, free standing, coating, fiber

## Abstract

The efficient dispersion of single-walled carbon nanotubes (SWCNTs) has been the subject of extensive research over the past decade. Despite these efforts, achieving individually dispersed SWCNTs at high concentrations remains challenging. In this study, we address the limitations associated with conventional methods, such as defect formation, excessive surfactant use, and the use of corrosive solvents. Our novel dispersion method utilizes the spontaneous charging of SWCNTs in a solvated electron system created by dissolving potassium in hexamethyl phosphoramide (HMPA). The resulting charged SWCNTs (c-SWCNTs) can be directly dispersed in the charging medium using only magnetic stirring, leading to defect-free c-SWCNT dispersions with high concentrations of up to 20 mg/mL. The successful dispersion of individual c-SWCNT strands is confirmed by their liquid-crystalline behavior. Importantly, the dispersion medium for c-SWCNTs exhibits no reactivity with metals, polymers, or other organic solvents. This versatility enables a wide range of applications, including electrically conductive free-standing films produced via conventional blade coating, wet-spun fibers, membrane electrodes, thermal composites, and core-shell hybrid microparticles.

## 1. Introduction

The debundling and individual dispersion of single-walled carbon nanotubes (SWCNTs) is a crucial step for the utilization of SWCNTs as the constituent material in various functional composites [1,2,3,4], templates [5,6,7], and electrically and thermally conductive films [8,9,10,11]. The advantages of SWCNTs, such as a high specific surface area and low percolation threshold [12,13], originate from the exceptionally high aspect ratio [14,15] and can be ideally realized when the SWCNT strands are individually separated prior to being processed into the targeted form. In order to debundle the SWCNTs, the Van der Waals interaction between the SWCNT strands must be overcome. The most common approach is the oxidation of SWCNTs, which can be achieved by treating the SWCNT bundles with chemical oxidants [16,17]. While the oxidation-based approach is effective for individual dispersions and achieving highly concentrated solutions, the degradation of intrinsic properties cannot be avoided. On the other hand, non-oxidative approaches, also adopted in the case of graphene exfoliation and utilization [18,19], utilize surfactants and polymeric dispersants combined with physically induced stresses, which may enable the debundling of SWCNTs without the deformation of the crystallinity. However, the dispersion process, using an intensive energy input, shows trade-off relations in the dispersion concentration and the length of individual SWCNT strands due to low debundling efficiency [20,21]. Moreover, such a process requires the excessive use of surfactants or dispersants, since the maximum concentration of individually dispersed SWCNTs is below 0.1 mg/mL when no surfactants are used [ref]. An alternative approach to effectively disperse SWCNTs is modulating the surface charge density of the SWCNTs to induce electrostatic repulsion between the SWCNT strands. The most widely known strategy is using superacids such as oleum or chlorosulfonic acid (CSA) to induce protonation on the surface of the SWCNTs [22,23]. An SWCNT concentration up to 10 wt% was reported, along with the development liquid-crystalline (LC) phases in accordance with the volume fraction and charge density change in the SWCNTs [23]. While the protonation leads to the positive charging of the SWCNTs, methods for negative charging were also reported [24,25,26]. The methods induce charge donation from alkali metals to the SWCNT bundles, which resemble the formation of graphite intercalation compounds [27]. When the negatively charged SWCNT bundles are exposed to aprotic organic solvents with sufficient polarity, the SWCNTs can be isolated in a facile manner without oxidation. The SWCNT dispersion methods based on the charging mechanism are especially advantageous in preserving the innate crystallinity along with possibility of achieving a high concentration. However, apparent limitations—such as the high corrosiveness of the superacids, the requirements for a low temperature [24,26] or introduction of mediators (i.e., benzene derivatives or crown ethers) [25], and the separately carried out steps for the charging and the dispersion—can lead to an inability or degraded performance when applying the dispersions for hybridization with other materials. 

In this research, a simple one-pot method for achieving a highly concentrated dispersion of a highly crystalline SWCNT using a solvated electron system is demonstrated. When an alkali metal is dissolved in ammonia and its derivatives, the free electrons from the alkali metal are stabilized by forming a complex-like structure with the bounding solvent molecules [28]. The stabilized electrons are termed “solvated electron”, which can be transferred to the conjugated structure in SWCNTs to increase the surface charge density [25,29]. Since the solvated electron system is in the liquid phase, the negatively charged SWCNTs could be directly dispersed in the same liquid medium if the selected solvent meets the criterion of sufficient surface tension [26,30]. Herein, we selected the hexamethyl phosphoramide (HMPA) as the liquid medium for the solvated electron system as well as the dispersion medium for the SWCNTs. We confirmed the spontaneous charging phenomenon occurring in SWCNT bundles in the electron solution, which led to the successful individual dispersion of SWCNTs, with the concentration reaching up to 20 mg/mL, using only magnetic stirring. The highly concentrated SWCNT dispersion exhibits an LC behavior and increased viscosity, which allow for the adaptation of simple and industry-friendly processes for the fabrication of robust film, fiber, and membrane, the electrical conductivity of which reach up to the order of 10^5^ S/m. We also demonstrate that the SWCNT dispersion can be used to fabricate a microparticle densely coated with SWCNTs, which can be a beneficial form of hybridized material for functional composites and energy electrode applications.

## 2. Materials and Methods

### 2.1. Materials

Bulk SWCNT powder was purchased from OCSiAl (Tuball, 94%, Luxembourg) and used without further purification. Potassium (99.9%), hexamethyl phosphoramide (HMPA, ACS Reagent grade), n-methyl pyrrolidone (NMP), and polyvinylidene fluoride (PVDF, MW 534 k) polystyrene (PS) microbeads (5–10 μm) were purchased from Sigma Aldrich (Saint Louis, MO, USA). HMPA was dried using a molecular sieve and magnesium chip for more than 24 h prior to usage. Ethanol and acetone were purchased from Samchun chemical (GR grade, Seoul, Republic of Korea). 

### 2.2. Fabrication of Electron Solution and Charged SWCNT Dispersion

**Figure 1:**
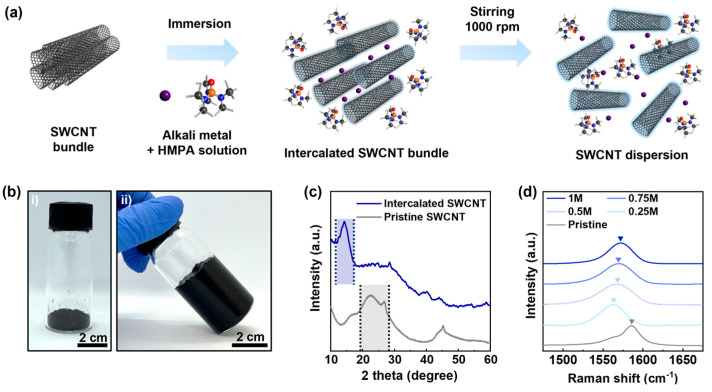
Charging behavior of SWCNTs in solvated electron system. (**a**) Schematic of c-SWCNT dispersion process. (**b**) Digital images of (i) p-SWCNT powder, (ii) c-SWCNT cake formed by the addition of electron solution. (**c**) XRD result of p-SWCNTs and c-SWCNTs. (**d**) Raman spectra of p-SWCNTs and c-SWCNTs exposed to electron solutions with different potassium concentration.

In order to form solvated electrons, bulk potassium was dissolved in HMPA with molar concentrations of 0.25 M, 0.5 M, 0.75 M, and 1 M. The mixture was magnetically stirred until the potassium was completely dissolved, typically requiring at least 3 h and additional time depending on the volume of the solution. In the case of the SWCNT dispersion, bulk potassium and SWCNTs were added together to HMPA, with the maximum concentration of SWCNTs in HMPA being 20 mg/mL. The mixture was then magnetically stirred at 1000 rpm for over 24 h to ensure that all SWCNTs mixed with the electron solution were completely debundled and formed a dispersion, which could be directly used without further treatment (Figure 1a). Both processes were performed under an inert atmosphere using a glove box.

### 2.3. Fabrication of SWCNT Film, Fiber, Membrane, Thermal Composite, and SWCNT-Coated Microparticle

#### 2.3.1. SWCNT Film Fabrication

The SWCNT film was fabricated using a simple coating process followed by drying and lift off. The SWCNT dispersion (20 mg/mL) was coated on aluminum foil using a typical film applicator (Doctor Blade). The coated film was dried on a hot plate at 100 °C for 1 h, followed by immersion in a water bath. The immersed SWCNT film was lifted off of the aluminum foil owing to the spontaneous diffusion of water into the interface of the film and the foil. After the lift off, the film was left to float for an additional hour to completely remove the residual potassium ion and HMPA inside the SWCNT film. The washed film was picked up using a PTFE mesh and was dried in an oven at 120 °C to obtain a free-standing SWCNT film.

#### 2.3.2. SWCNT Fiber Fabrication

The SWCNT fiber was fabricated through a wet-spinning process. The SWCNT dispersion (20 mg/mL) was used as the dope, and deionized (DI) water or a mixture of DI water and ethanol were used as the coagulant. In detail, the SWCNT dispersion was charged into a syringe and discharged into the rotating coagulant bath using a syringe pump at the rate of 0.3 mm/min. The size of the nozzle was 20 G, and the rotating speed of the coagulation bath was approximately 60 rpm. The spun fiber was moved to fresh DI water and kept for 10 min before winding and drying at 80 °C.

#### 2.3.3. SWCNT Membrane Fabrication

The SWCNT membrane was fabricated using vacuum filtration of the SWCNT dispersion. The SWCNT dispersion (20 mg/mL) was diluted with NMP using a vortex mixer to obtain an SWCNT dispersion with a concentration of 1 mg/mL. Then, 10 mL of the diluted dispersion was vacuum filtrated over an anodize alumina (AAO) filter membrane and washed with 500 mL of DI water and 250 mL of ethanol. The SWCNT membrane was dried in oven at 120 °C, and the SWCNT membrane was peeled off from the AAO filter. 

#### 2.3.4. SWCNT Thermal Composite Fabrication

The SWCNT-based thermal composite was fabricated by solution mixing of the SWCNT and PVDF, followed by the mold casting process. First, the PVDF powder was dissolved in NMP to a concentration of 100 mg/mL using magnetic stirring at 60 °C. Afterwards, the SWCNT dispersion (20 mg/mL) was mixed with the PVDF solution so that the weight ratio of the SWCNT compared to the PVDF was 0.25 wt%, 0.5 wt%, 0.75 wt%, and 1 wt%, using a planetary mixer (Thinky) at 2000 rpm for 15 min. The mixture was coated onto aluminum foil and degassed for 30 min in a vacuum oven, followed by the drying process at 130 °C for 8 h. The fabricated composite film was lifted off of the Al foil through immersion in DI water, analogous to the SWCNT film fabrication process. The composite film was then dried in oven to remove the moisture on the surface.

#### 2.3.5. SWCNT-Coated Microparticle Fabrication

The SWCNT-coated microparticle was fabricated by drop casting the mixture of the SWCNT dispersion and PS microbeads. Here, 100 mg of PS microbeads were added to the 10 mL of SWCNT dispersion (1–5 mg/mL). After vortex mixing for 1 min, the mixture was drop casted into an acetone bath using a micropipette. The coagulated particle that sedimented at the bottom of the acetone bath was retrieved using vacuum filtration and a drying process analogous to the membrane fabrication process.

### 2.4. Characterizations and Measurements

#### 2.4.1. Analysis of SWCNTs Exposed to the Electron Solution

The SWCNTs exposed to the solvated electron system were analyzed using X-ray diffraction analysis (XRD, X’Pert PRO MPD, Panalytical) and Raman spectroscopy (ANT-QNT, Nt-mdt). In order to prepare the samples, pristine SWCNT powder was packed into a homemade holder, which could be sealed using a cover glass and sealant after the addition of the electron solution. The reference pristine sample was analyzed without exposure to the electron solution. 

#### 2.4.2. Morphology and Crystallographic Analyses

The morphological and crystallographic characterizations of the dispersed SWCNTs and the SWCNT film, fiber, membrane, and hybrid microparticles were performed using polarized optical microscopy (POM, Nikon), scanning electron microscopy (SEM, S-4800, Hitachi), atomic force microscopy (AFM, NX 10, Park systems), X-ray photoelectron spectroscopy (XPS, K-Alpha, Thermo Fischer), Raman spectroscopy, and transmission electron spectroscopy (TEM, TALOS F200X, FEI). For the POM analysis, approximately 10 μL of the SWCNT dispersion was casted and sandwiched between two cover glasses, which were carefully dried at 120 °C prior to the sampling. AFM samples were prepared by drop casting a drop of the SWCNT dispersion diluted to a concentration of 1 mg/mL onto to the surface of DI water filled in a 100 mL beaker. The SWCNT thin film floating over the DI water was lifted-off using a SiO_2_ wafer and dried at 120 °C for 3 h before the AFM analysis. The samples for SEM, XPS, and Raman spectroscopy were prepared by using carbon tape to place the free-standing SWCNT film, fiber, membrane, and hybrid microparticles onto a SiO_2_ wafer chipped to a size of 1 cm in length and width. The TEM sample was prepared using the same protocol for the AFM sampling, except that the concentration of the SWCNT dispersion was lowered to 0.1 mg/mL, and the SWCNT was picked up using a lacey carbon-coated copper TEM grid (mesh 300).

#### 2.4.3. Analysis of Electrical Conductivity of SWCNT Thin Film

The electrical conductivity of the fabricated SWCNT film was analyzed by measuring the sheet resistance and the thickness of the respective samples. Five different points in each sample were analyzed using a four-point probe resistance tester (MCP-T610, Mitsubishi) and a thin film-thickness gauge (547–400 s, Mitsutoyo). The conductivity of the sheet was calculated by using the Equation (1):

σ = 1/ρ = 1/R_sheet_ × H (1)

Here, σ is the conductivity, ρ is the resistivity, R_sheet_ is sheet resistance, and H is the thickness of the sample.

#### 2.4.4. Electrochemical Characterization

The prepared SWCNT films were used as cathodes for lithium-ion capacitors. The mass loadings of the cathodes were 0.1–0.2 mg cm^−2^. Coin half cells were assembled in an Ar-filled glove box. The Li metal sheets (thickness = 0.5 mm) and the mixture of ethylene carbonate and dimethyl carbonate (7:3 = *v*:*v*) containing 1 M lithium hexafluorophosphate (LiPF6) were used as reference electrodes and electrolytes, respectively. The galvanostatic charge/discharge and rate capability characteristics of half cells were evaluated using a potentiostat/galvanostat instrument (VMP3, Bio-Logics). The precyling test was conducted in a voltage window of 1.5–4.5 V (vs Li/Li+); the charge and discharge current density for the precycling test was 0.1 A g^−1^.

## 3. Results and Discussion

### 3.1. Charging of SWCNTs in Solvated Electron System

Figure 1a is the schematic for the dispersion process of SWCNTs through the spontaneous charging phenomenon in a solvated electron system using potassium and HMPA. When the pristine SWCNT (p-SWCNT) powders are immersed in the electron solution, the SWCNTs are negatively charged, which induces the intercalation of potassium ion and HMPA molecules into the inter-strand spaces of the charged SWCNTs (c-SWCNTs). The intercalation will result in a significant increase in the inter-strand distances, allowing the c-SWCNTs to be debundled with only a low-energy agitation using magnetic stirring. Figure 1b shows the digital images of the p-SWCNT powder and the swelled-up cakes of c-SWCNTs formed by the addition of the electron solution. It should be noted that the electron solutions are instantly absorbed into the SWCNT bundles, forming a cake with no visible phase separation of liquid and solid components, as shown in Figure 1b(ii). The XRD result in Figure 1c confirms the intercalation phenomenon by revealing the formation of a new peak centered at 13.4° in the case of intercalated c-SWCNTs, which corresponds to the increased inter-strand spacing compared to the p-SWCNTs, with their main diffraction peaks located in between 20° and 28°. The Raman spectra analysis of the intercalated c-SWCNTs in Figure 1d shows that the G peaks of the c-SWCNTs are red-shifted, which indicates negative charging [31]. It is noteworthy that the degree of the peak shift did not vary a lot, regardless of the molar concentration of potassium in the solvated electron system. The reason for a small variance may be the local charge compensation occurring between the negatively charged c-SWCNTs and the intercalated cationic complex species. As the c-SWCNTs have a higher degree of surface charge, a larger number of cationic species can be intercalated, resembling the staging behavior in graphite intercalation compounds. Such prediction is partly proved by dispersing the intercalated c-SWCNTs in ethanol. The ethanol partially contains moisture, which quenches the dispersed c-SWCNTs, resulting in the aggregation, as shown in Figure S1. The degree of the peak shift showed a tendency to slightly decrease as the molar concentration of the K ion in the electron solution increased. Currently, we are unsure of the exact origin of this phenomenon, but we suspect the different amount of intercalated species and the resulting inter-strand spacing may change the electron–phonon interference in the SWCNT bundles [32]. Nevertheless, as the molar ratio of the potassium is higher in the initial condition, the c-SWNTs tend to be dispersed into finer bundles, implying that the larger number of inter-strand spaces are occupied by the intercalant species. Therefore, to obtain individually dispersed c-SWCNTs, it is preferable to disperse the SWCNTs in an electron solution with a higher potassium concentration (i.e., 1 M in this work). 

### 3.2. Defect-Free Dispersion of Charged SWCNTs

**Figure 2:**
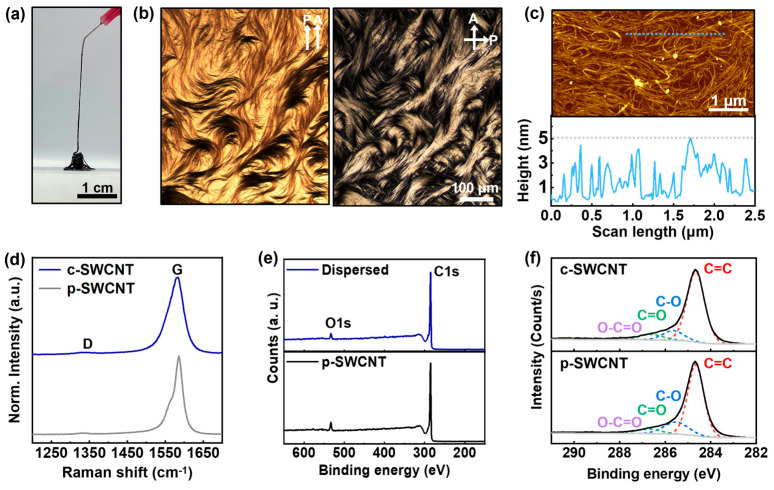
Characterization of c-SWCNT dispersion. (**a**) Digital image of c-SWCNT dispersion (20 mg/mL). (**b**) POM images of c-SWCNT dispersion (20 mg/mL): bright field image (**left**); dark field image (**right**). (**c**) AFM image of c-SWCNT sampled on SiO2 wafer (**top**); height profile corresponding to the dotted line in the AFM image (**bottom**). (**d**) Normalized Raman spectra of c-SWCNT and p-SWCNT films. (**e**) XPS survey of c-SWCNT and p-SWCNT films. (**f**) C1s scan of c-SWCNT and p-SWCNT films.

Figure 2a is the digital image of the c-SWCNT dispersion fabricated with an electron solution with a potassium concentration of 1 M. The c-SWCNT dispersion extruded from the nozzle piles up on the substrate, indicating its high viscosity. The increase in viscosity is a typical phenomenon observed in dispersions containing nanomaterials with a high aspect ratio [33], implying that the c-SWCNTs are effectively dispersed in the electron solution. The dispersion state of the c-SWCNTs can be observed by using POM. The POM images of the c-SWCNT dispersion shown in Figure 2b reveal that the dispersed c-SWCNTs exhibit an LC behavior in resemblance to the observations in previous works using superacids [23]. The LC behavior of c-SWCNTs can be achieved only when the SWCNTs can be individually dispersed at a sufficiently high concentration, when no additives are used. The individual dispersions of c-SWCNTs were further confirmed using AFM. Several points with height values around 1.2 nm were detected in the AFM result in Figure 2c, which corresponds to the summation of a single c-SWCNT strand diameter (i.e., 0.9 nm), measured with TEM (Figure S2), and the distance between the SWCNT and the SiO_2_ substrate [34]. The results indicate that single SWCNT strands can be isolated even at a high concentration, with the maximum diameter of the bundles formed during the sampling process being less than 5 nm. The superb dispersion efficiency also allows for the preservation of crystallinity. The c-SWCNTs require only a mild dispersion process such as magnetic stirring, which limits the generation of defects in contrary to high-energy dispersion techniques such as high-pressure homogenization. The Raman spectra of the c-SWCNT film in Figure 2d show no visible change in the D peak compared to that of the p-SWCNT film, indicating that no defects are formed during the dispersion process. The XPS survey and C1s scan in Figure 2e,f also show that no defect formation and oxidation occur in c-SWCNTs. The oxygen content in the c-SWCNT sample was 3.74 at%, which is slightly less than in the p-SWCNT sample (5.68 at%). The decreased oxygen is possibly due to the strong reducing property of the electron solution, as evidenced by the decreased hydroxyl bond (C-O, 285.6 eV) in c-SWCNTs compared to the p-SWCNTs.

### 3.3. Fabrication of Free-Standing Film and Fiber Using c-SWCNT Dispersion

**Figure 3:**
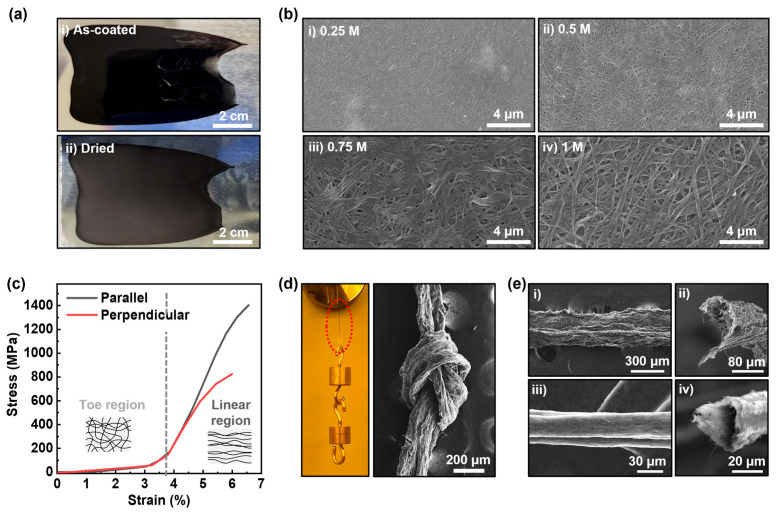
Free-standing film and fiber fabricated with c-SWCNT dispersion. (**a**) Digital images of (**i**) c-SWCNT dispersion coated on Al foil using blade coating, (**ii**) dried c-SWCNT film. (**b**) SEM images of c-SWCNT film fabricated with c-SWCNT dispersion with K concentrations of (**i**) 0.25 M, (**ii**) 0.5 M, (**iii**) 0.75 M, and (**iv**) 1 M. (**c**) Stress–strain curve of the c-SWCNT film (0.25 M). (**d**) Digital image and SEM image of the wet-spun c-SWCNT fiber. (**e**) SEM images of wet-spun c-SWCNT fiber fabricated using (**i**) DI water coagulant, (**ii**) DI water–ethanol solution coagulant.

The LC behavior of the c-SWCNT dispersion, combined with the non-corrosive properties of HMPA, allow for its application in a variety of conventional fabrication process used in industries, such as blade coating or wet spinning. Figure 3a shows the digital images of c-SWCNT film fabricated by coating of the c-SWCNT dispersion on an Al foil. The c-SWCNT dispersion exhibits sufficient viscosity and adhesion to form a smooth layer over the foil, the morphology of which is preserved after the drying process. The c-SWCNT film can be easily separated from the foil by simply immersing the sample into DI water. When the sample is immersed, the water molecules diffuse into the interface of the film and the foil, where the K ions remaining inside the c-SWCNT film react with the water to generate hydrogen gas, which form bubbles, leading to the separation of the c-SWCNT film and the Al foil. The Figure 3b shows the surface image of the films fabricated using c-SWCNT dispersions with different K ion concentrations. The analysis reveals that the c-SWCNT dispersion with a higher K ion concentration shows the tendency to form a c-SWCNT film with larger pores and a higher degree of bundling. Such a tendency can be attributed to the different amounts of potassium hydroxide salt formed during the drying process and the hydrogen gas formation reaction occurring within the film, depending on the initial K ion concentration. The average electrical conductivity of the c-SWCNT film reached up to 600,000 S/m (Table 1), the value of which matches the preliminary results achieved in densely packed SWCNT fibers fabricated via LC-phase spinning [23]. A comparative analysis on the electrical conductivity of a membrane film fabricated with p-SWCNTs was shown to be approximately 22,300 S/m (Figure S3). The low electrical conductivity value of the p-SWCNT membrane is possibly the result of an overestimated thickness value due to the existence of thick bundles and the low packing density of the SWCNTs in the membrane. The packing density of the c-SWCNT films should also affect their mechanical strength, since it is dominated by the Van der Waals interaction at the interfaces of the packed c-SWCNTs. Figure 3c is the tensile test result of the c-SWCNT film fabricated with a K-ion concentration of 0.25 M. The stress–strain curve of the c-SWCNT film exhibits two regions showing different types of strain behaviors. The curve shows a linear increase up to a strain of 3% and begins to show an exponential increase until the strain reaches 3.7%. After 3.7%, the curve shows a common shape corresponding to elastic and plastic deformations of a bulk material. The reason for the different mechanical behaviors at different strain regimens is possibly attributed to the network structure of the c-SWCNT film, which resembles the fibrous structure of biological tissues, showing similar tensile behavior [35]. In the first region (i.e., strain < 3.7%), the SWCNTs in the film rearrange to align in the direction of induced tension, relieving the structural stress in the film. After the rearrangement ends, the film shows a bulk-like behavior, which is due to the interfacial bonding between the SWCNTs, possibly relaxed by the slipping phenomenon at a higher strain regimen [36]. It should be noted that the SWCNTs in the film are not completely aligned by the induced tension, as depicted by the different yield strengths between two different tensile directions. When the tension is applied parallel to the direction of c-SWCNT coating, the yield strength reaches 1.4 GPa, while it reaches only 0.8 GPa when the tension is applied perpendicular to the coating direction. The result suggests that the c-SWCNTs are pre-aligned to a certain degree due to the inducement of shear force during the coating process. Using the shear-induced alignment property, the c-SWCNT dispersion can be utilized as a dope for the wet-spinning process to fabricate SWCNT fibers. The SWCNTs are intrinsically hydrophobic, while the HMPA is miscible with water, implying that it is possible to apply a conventional wet-spinning mechanism involving the injection of dope solution into a coagulation bath filled with antisolvent. Figure S4 shows the instrumental configuration for wet-spun c-SWCNT fiber fabrication. When the c-SWCNT dope is injected into the coagulation bath, the HMPA molecules diffuse out of the dope, and the remaining c-SWCNTs aggregate to form fibers. Figure 3d shows the digital image and SEM image of the exemplary c-SWCNT fiber fabricated by using DI water as the coagulant. A single strand of fiber with width of 200 μm showed mechanical robustness sufficient to withstand the weight up to 2 g and to form a knot. The SEM images in Figure 3e are the comparative images of the fibers spun in DI water (Figure 3e(i,ii)) and a mixture of DI water and ethanol (80:20 = *v*:*v*) (Figure 3e(iii,iv)). The SEM images show that the surface texture of the spun fiber becomes smoother in the case of the ethanol solution coagulant and that the cross-sectional shape of the spun fiber is more circular compared to that of the fiber spun in DI water. Moreover, the widths of the fibers spun using different coagulants vary significantly due to the differences in the cross-sectional morphology as well as the packing density of the SWCNTs in the fiber. The change in the fiber morphology is due to the change in the diffusion rate of the HMPA molecules, which becomes slower in ethanol compared to water. When the diffusion rate of the solvent is too high, the solute in the dope has an insufficient amount of time to densely pack during the fiber formation, resulting in an increased surface roughness as well as an anisotropic cross-sectional morphology. Further optimization for the spinning conditions will be conducted to increase the packing density of the fiber and relevant properties.

### 3.4. Versatile Applications of c-SWCNT Dispersion

**Figure 4:**
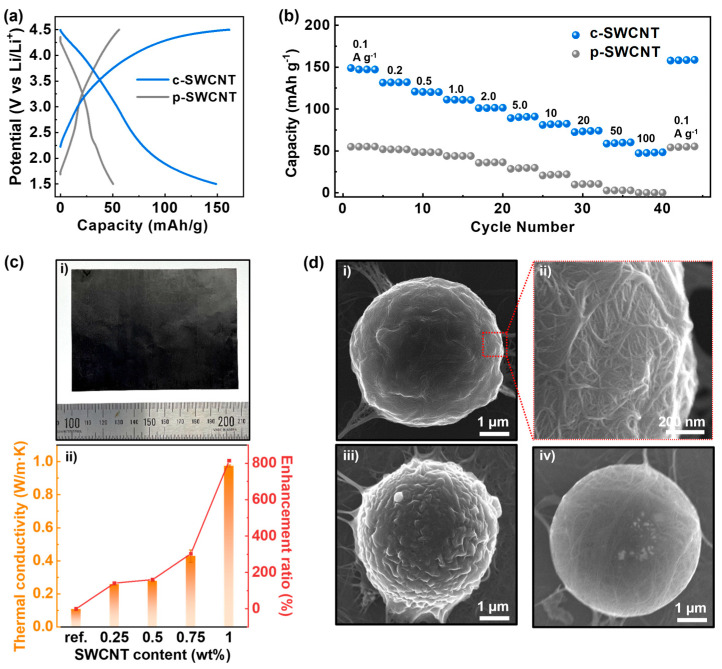
Versatile applications of c-SWCNT. (**a**) Galvanostatic charge–discharge profile of c-SWCNT and p-SWCNT cathodes (current density = 0.1 A g^−1^). (**b**) Rate capability comparison of c-SWCNT and p-SWCNT cathodes. (**c**) (**i**) Digital image of SWCNT-PVDF composite (1 wt%); (**ii**) thermal enhancement in c-SWCNT-PVDF composite. (**d**) SEM images of hybridized c-SWCNT-PS microsphere fabricated with c-SWCNT dispersions with concentrations of (**i**,**ii**) 2 mg/mL, (**iii**) 5 mg/mL, and (**iv**) 1 mg/mL.

A strong advantage of the c-SWCNT dispersion is the ability to be directly mixed or hybridized with different solvents, polymers, and particles owing to its non-corrosive nature. Figure 4a,b show the electrochemical performance of a porous membrane electrode composed of c-SWCNT applied in a Li-ion battery. As explained in the Materials and Methods section, the c-SWCNT dispersion was diluted using NMP and then vacuum filtrated to form a film with pores that had diameters generally smaller than 100 nm, as shown in Figure S4. The higher gravimetric capacity of the c-SWCNT electrode compared to p-SWCNT electrode suggests that the degree of debundling is higher in the c-SWCNT electrode, resulting in a larger amount of accessible area for the Li ions. In addition to the increased capacity, the higher degree of debundling also allows for stability at a high current condition. The Figure 4b shows the capacity under different current density conditions ranging from 0.1 A g^−1^ up to 100 A g^−1^. It is shown that the capacity of the c-SWCNT electrode charged at a current density of 100 A g^−1^ is comparable to that of the p-SWCNT electrode at a current density of 0.1 A g^−1^. Moreover, the c-SWCNT electrode shows excellent recovery of its discharge capacity after a high c-rate (100C) test. The combination of a high electrical conductivity and high porosity owing to the high degree of debundling allows for the fast diffusion of the Li^+^ and PF6^-^ ions onto the surface of SWCNTs, resulting in a reduced charge-transfer resistance at the interface between the electrode and electrolytes during the charging/discharging cycles. The c-SWCNTs can also be mixed with polymer solutions to fabricate thermal composites. The c-SWCNT dispersion was mixed with PVDF solution and casted by blade coating, followed by a drying process. Figure 4c shows the increase in the in-plane thermal conductivity of the c-SWCNT-PVDF composite film with different c-SWCNT contents. As the filler content becomes larger than 0.5 wt%, the thermal conductivity begins to increase exponentially, and at 1 wt%, the thermal enhancement ratio reaches near 800% compared to the pristine PVDF film. Another strategy to hybridize the c-SWCNT with other materials is to form a coating layer on the surface of microparticles. When PS microspheres are mixed into the c-SWCNT dispersions, the c-SWCNTs can adhere to the surface of the microsphere owing to the π–π interaction between the conjugate structure in PS and the SWCNTs. After mixing, the dispersion pipetted out from the mixed solution was drop casted into the acetone bath to induce the coagulation process, which results in the formation of a densely packed overlayer of c-SWCNTs. As observed in the SEM image of the hybridized c-SWCNT-PS microsphere in Figure 4d, the coating process results in the formation of a partially wrinkled surface composed of packed c-SWCNTs. The morphology and density of the c-SWCNTs in the coated layer can vary depending on the relative ratio of the PS microspheres mixed into the c-SWCNTs dispersion, which implies that the optimization of the hybridization ratio is possible, depending on the targeted application.

## 4. Conclusions

In summary, the effective debundling and individual dispersion of SWCNTs by the using charging phenomenon in a solvated electron system composed of potassium and HMPA has been successfully demonstrated. The dispersion of c-SWCNTs could be fabricated with high concentrations reaching up to 20 mg/mL without the use of any surfactant, while the mild process resulted in no formation of additional defects. The liquid-crystalline behavior of the c-SWCNTs originating from the combination of effective dispersion and a sufficiently high concentration, combined with the non-corrosive nature of HMPA, allowed for the direct application of the c-SWCNT dispersion in conventional blade-coating and wet-spinning processes. Moreover, the dispersion could be mixed with other organic solvents or polymeric materials for the formation of a membrane electrode in a supercapacitor, thermal composites, and SWCNT-coated hybrid microparticles. The optimization and stabilization of the processes for the respective applications remain as the future tasks, yet the defect-free and surfactant-free natures and the versatility of the c-SWCNT dispersion will provide a strong basis for developing high-performance applications using SWCNTs as the functional additives.

## Figures and Tables

**Table 1:** Sheet resistance and electrical conductivity of the c-SWCNT film (K concentration = 0.25 M).

Point	Thickness (μm)	Sheet Resistance ^1^ (Ω/□)	Electrical Conductivity (S/m)
1	4	0.349	716,332
2	4	0.408	612,745
3	4	0.744	336,021
4	4	0.201	1,243,781
5	4	0.436	573,394
Avg.	4	0.4276	696,455

^1^ The digital images of the sheet resistance measurement results are shown in Figure S3.

## Data Availability

Data are contained within the article.

## Supplementary Materials

The following supporting information can be downloaded at: https://www.mdpi.com/article/10.3390/nano14131094/s1, Figure S1: Dispersion in ethanol vs. K-HMPA molar concentration; Figure S2: TEM images of SWCNT strand; Figure S3: Digital images of sheet resistance measurement results; Figure S4: Schematic of basic setup for wet-spinning of c-SWCNT fiber; Figure S5: SEM image of c-SWCNT membrane electrode.
