# Supplementary material for: Ultra-Mild Fabrication of Highly Concentrated SWCNT Dispersion Using Spontaneous Charging in Solvated Electron System
Source: Nanomaterials (Basel). 2024 Jun 26;14(13):1094. doi: 10.3390/nano14131094 (PMC11243719; doi:10.3390/nano14131094)
Supplement: Supplementary file 1 [file nanomaterials-14-01094-s001.zip › nanomaterials-3072742-supplementary.pdf]

Supplementary information

Ultra-mild Fabrication of Highly Concentrated Surfactant-free, Defect-free SWCNT Dispersion using Solvated Electron System

Junho Shin [1,2], Jung Hoon Kim [1], Jungeun Lee [1], Sangyong Lee [1], Jong Hwan Park [1], Seung Yol Jeong [1], Hee Jin Jeong [1], Joong Tark Han [1], Seon Hee Seo [1], Seoung-Ki Lee [2,*] and Jungmo Kim [1,*]

[1] Nano Hybrid Technology Research Center, Korea Electrotechnology Research Institute (KERI), Changwon 51543, Republic of Korea; sinjh@keri.re.kr (J.S.); kjh40210@keri.re.kr (J.H.K.); eeueu@keri.re.kr (J.L.); tkddyd@keri.re.kr (S.L.); jhpark79@keri.re.kr (J.H.P.); syjeong@keri.re.kr (S.Y.J.); wavicle11@keri.re.kr (H.J.J.); jthan@keri.re.kr (J.T.H.); seosh@keri.re.kr (S.H.S.)

[2] School of Material Science and Engineering, Pusan National University, Busan 46241, Republic of Korea

* Correspondence: ifriend@pusan.ac.kr (S.-K.L.); jungmokim@keri.re.kr (J.K.); Tel.: +82-51-510-2999 (S.-K.L.); +82-55-280-1639 (J.K.)

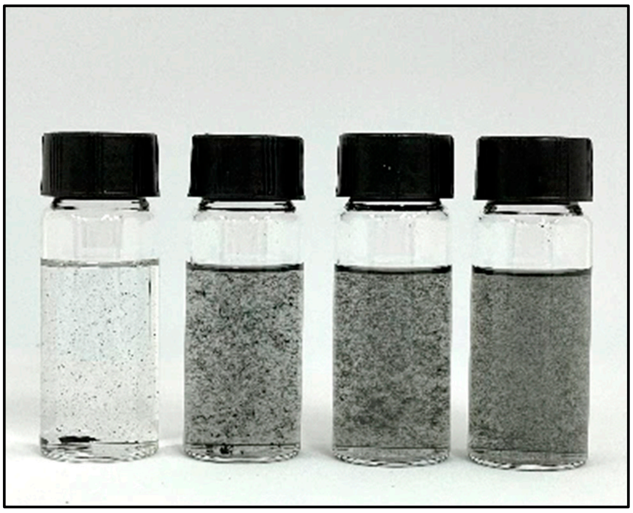

Figure S1. Dispersion in ethanol vs K-HMPA molar concentration. Dilute ethanol dispersion of c-SWCNTs fabricated in K-HMPA solution with K molar concentration of 0.25 M, 0.5 M, 0.75 M, 1 M (Left to right)

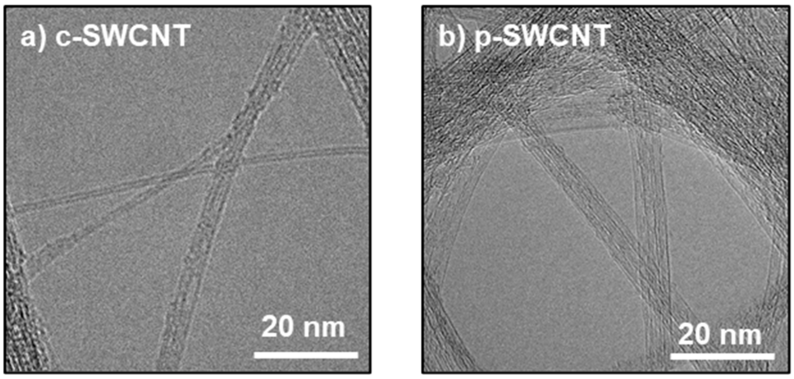

Figure S2. TEM image of SWCNT strand. (a) c-SWCNT. (b) p-SWCNT

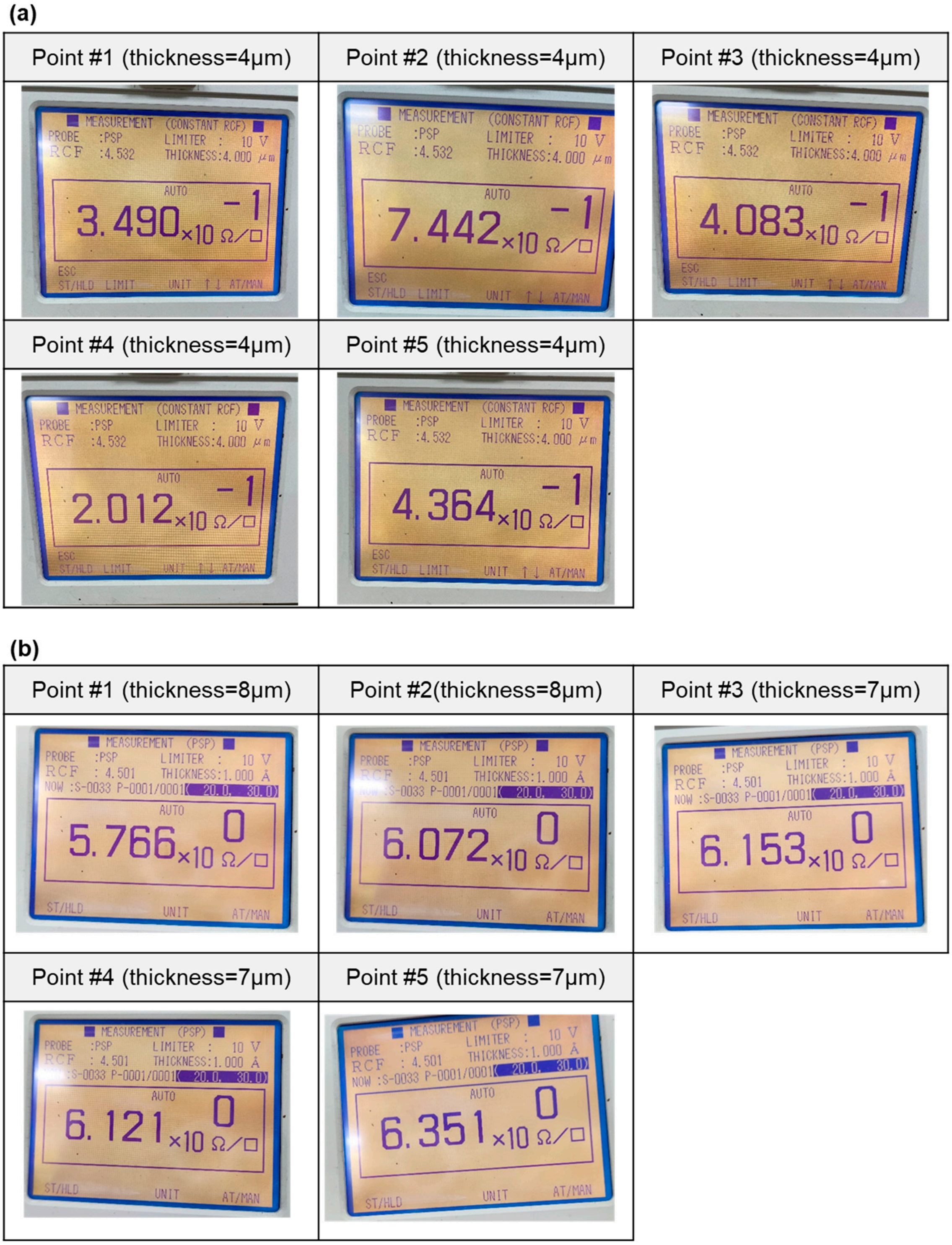

Figure S3. Digital images of sheet resistance measurement results. (a) Sheet resistance of c-SWCNT film, (b) Sheet resistance of p-SWCNT film

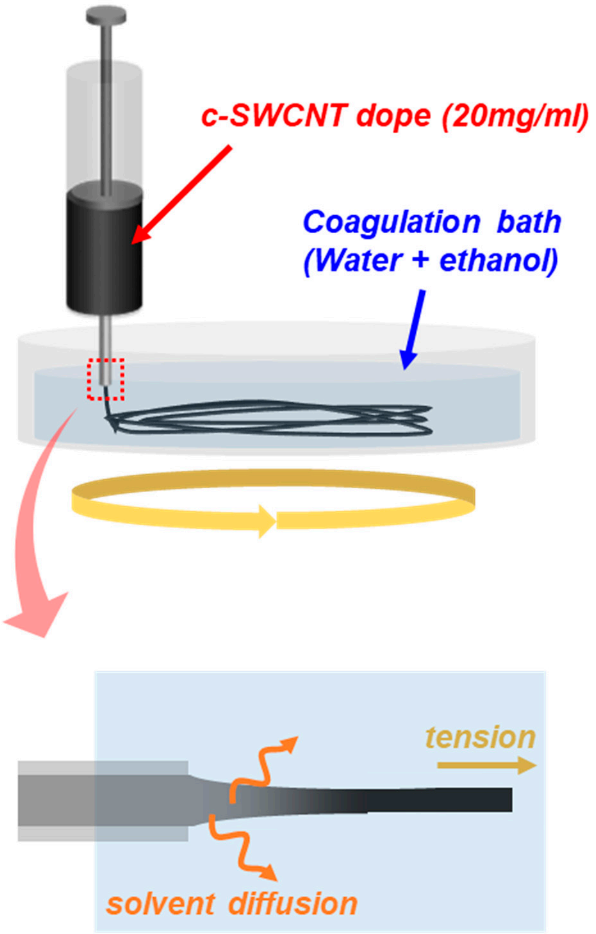

Figure S4. Schematic of basic setup for wet-spinning of c-SWCNT fiber.

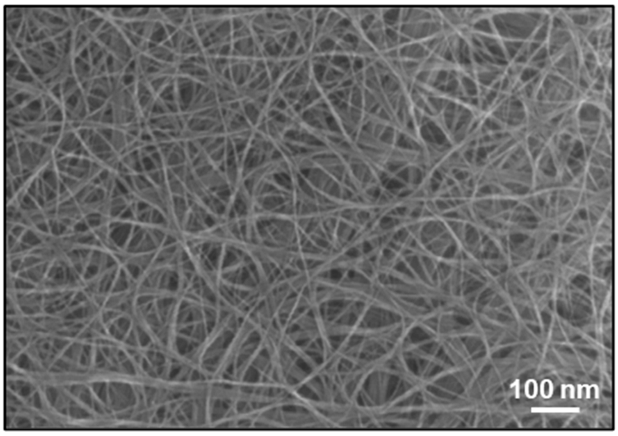

Figure S5. SEM image of c-SWCNT membrane electrode
